# A quantitative evaluation method utilizing the homology concept to assess the state of chromatin within the nucleus of lung cancer

**DOI:** 10.1038/s41598-023-46213-w

**Published:** 2023-11-09

**Authors:** Yuhki Yokoyama, Kazuki Kanayama, Kento Iida, Masako Onishi, Tadasuke Nagatomo, Mayu Ito, Sachiko Nagumo, Kunimitsu Kawahara, Eiichi Morii, Kazuaki Nakane, Hirofumi Yamamoto

**Affiliations:** 1 Department of Molecular Pathology, Division of Health Sciences, Graduate School of Medicine, Osaka University, 1-7, Yamadaoka, Suita, Osaka 565-0871 Japan; 2 Department of Clinical Nutrition, Suzuka University of Medical Science, 1001-1 Kishioka, Suzuka, Mie 510-0293 Japan; 3 Department of Pathology, Osaka Habikino Medical Center, 3-7-1, Habikino, Habikino, Osaka 583-8588 Japan; 4 Department of Diagnostic Pathology, Osaka University Hospital, 2-15 Yamadaoka, Suita, Osaka 565-0871 Japan; 5 Division of Pathology for Regional Communication, Graduate School of Medicine, Kobe University, 7-5-1 Kusunoki-Cho, Chuo-Ku, Kobe City, Hyogo 650-0017 Japan

**Keywords:** Cancer, Cell biology, Computational biology and bioinformatics, Pathogenesis

## Abstract

Homology is a mathematical tool to quantify "the contact degree", which can be expressed in terms of Betti numbers. The Betti numbers used in this study consisted of two numbers, b0 (a zero-dimensional Betti number) and b1 (a one-dimensional Betti number). We developed a chromatin homology profile (CHP) method to quantify the chromatin contact degree based on this mathematical tool. Using the CHP method we analyzed the number of holes (surrounded areas = b1 value) formed by the chromatin contact and calculated the maximum value of b1 (b1MAX), the value of b1 exceeding 5 for the first time or Homology Value (HV), and the chromatin density (b1MAX/ns^2^). We attempted to detect differences in chromatin patterns and differentiate histological types of lung cancer from respiratory cytology using these three features. The HV of cancer cells was significantly lower than that of non-cancerous cells. Furthermore, b1MAX and b1MAX/ns^2^ showed significant differences between small cell and non-small cell carcinomas and between adenocarcinomas and squamous cell carcinomas, respectively. We quantitatively analyzed the chromatin patterns using homology and showed that the CHP method may be a useful tool for differentiating histological types of lung cancer in respiratory cytology.

## Introduction

Lung cancer is the leading cause of cancer-related deaths worldwide^1, 2^ and various risk factors, such as smoking^3^, are implicated in increased risk^4^. Based on differences in treatment strategies, lung cancer is divided into two major types: non-small cell carcinoma and small cell carcinoma. However, non-small cell carcinoma can be further divided into squamous cell carcinoma and adenocarcinoma due to the development of molecular targeted therapies^5^. Gene mutations such as EGFR and ALK in non-squamous cancers, mainly adenocarcinomas, can be assayed and if positive, molecular targeted therapies including gefitinib and crizotinib can be prescribed^6, 7^. For mutation negative patients, platinum-based chemotherapy is performed. However, no effective molecular targeted therapy has been developed for squamous cell carcinoma or small cell carcinoma, so in these cases platinum-based chemotherapy is also recommended^8^. Lung cancer screening is mainly performed by chest X-ray, and sputum cytology is recommended for patients over the age of 50 with a smoking index of 600 or higher in Japan. If abnormal shadows are observed on the X-ray images, the patient is referred for a biopsy of the lesion, which is processed via cytology for the diagnosis.

The one clinical issue for the use of cytology is a chronic shortage of cytologists to determine the diagnosis. Increasing the number of cytologists requires time for training. Therefore, the development of an automated cytology diagnostic support system would reduce the burden on cytologists. An example of automated cytology is the LC-1000 exfoliating cell analyzer (Sysmex, Hyogo, Japan) for cervical cancer screening^9^. In this system, the DNA of each cell is stained with a fluorescent dye, and analyzed by flow cytometry. The scattered light signal is quantified as the size of the cell, and the fluorescent signal is quantified as the size of the nucleus and the amount of nuclear DNA. Based on these parameters, this system can detect the signature of cellular malignancy and proliferative activity such as a high N/C ratio (Nuclear/Cytoplasm ratio). Another example is the ThinPrep Imaging System Duo (Hologic, Marlborough, Massachusetts, USA). Although it is not fully automated, the system can scan each specimen and identify 22 fields of view, which contain suspected malignant cells^10, 11^. In comparison to the LC-1000 system, the ThinPrep Imaging System Duo uses imaging technology. After the images containing the suspected malignancies are identified, a cytologist determines the final diagnosis. An automated cytological diagnosis is possible in cervical cancer because the number of specimens is large and morphological differences such as a high N/C ratio between normal cells and cancer cells are clearer than other cancer types. However, the development of an automated system for respiratory cytology is more challenging.

Artificial Intelligence (AI) is a possible approach to aid in the development of automated cytology. Once a large imaging dataset and corresponding clinical information are supplied to the machine learning algorithms, common patterns can be identified and linked to clinical information. This approach has been used previously for x-ray images because of the normal uniformity of an X-ray image^12–14^. However, the images obtained from respiratory cytology present a challenge to the machine learning algorithms, because the patterns of nuclear shape and staining (referred to as the chromatin pattern) are complicated and have a high degree of variability. In particular, the chromatin pattern differs among the various histological types including squamous cell carcinoma, adenocarcinoma and small cell carcinoma, and can also vary within the same histological type. Therefore, an alternative strategy is needed to develop a method for automated respiratory cytology.

To accomplish this goal, we focused on a homology-based methodology (i.e. the calculation of Betti numbers) to quantitively evaluate the chromatin pattern of lung non-cancerous cells versus cancer cells. We previously reported that the colonic, prostate and lung tumor areas on digital images of H & E staining could be identified based on Betti numbers^15–18^. In this study, we investigated whether this homology-based method could be developed for an automated respiratory cytology application.

## Results

### Comparison of the homology values for lung cancer cells versus non-cancerous cells

**Figure 1:**
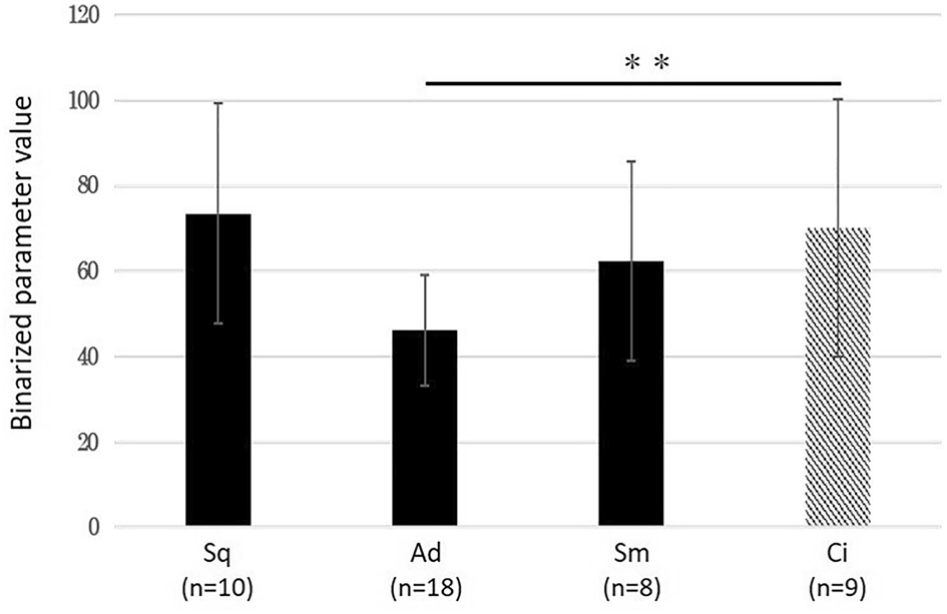
Comparison of the HV across histological types. There was a significant difference between non-cancerous and adenocarcinoma (Student's t test; **P < 0.01), but no significant differences between non-cancerous vs squamous carcinoma and non-cancerous vs small cell carcinoma were found. Data are shown as the mean ± SD. *Sq* squamous cell carcinoma, *Ad* adenocarcinoma, *Sm* small cell carcinoma, *Ci* ciliated columnar epithelial cell.

**Figure 2:**
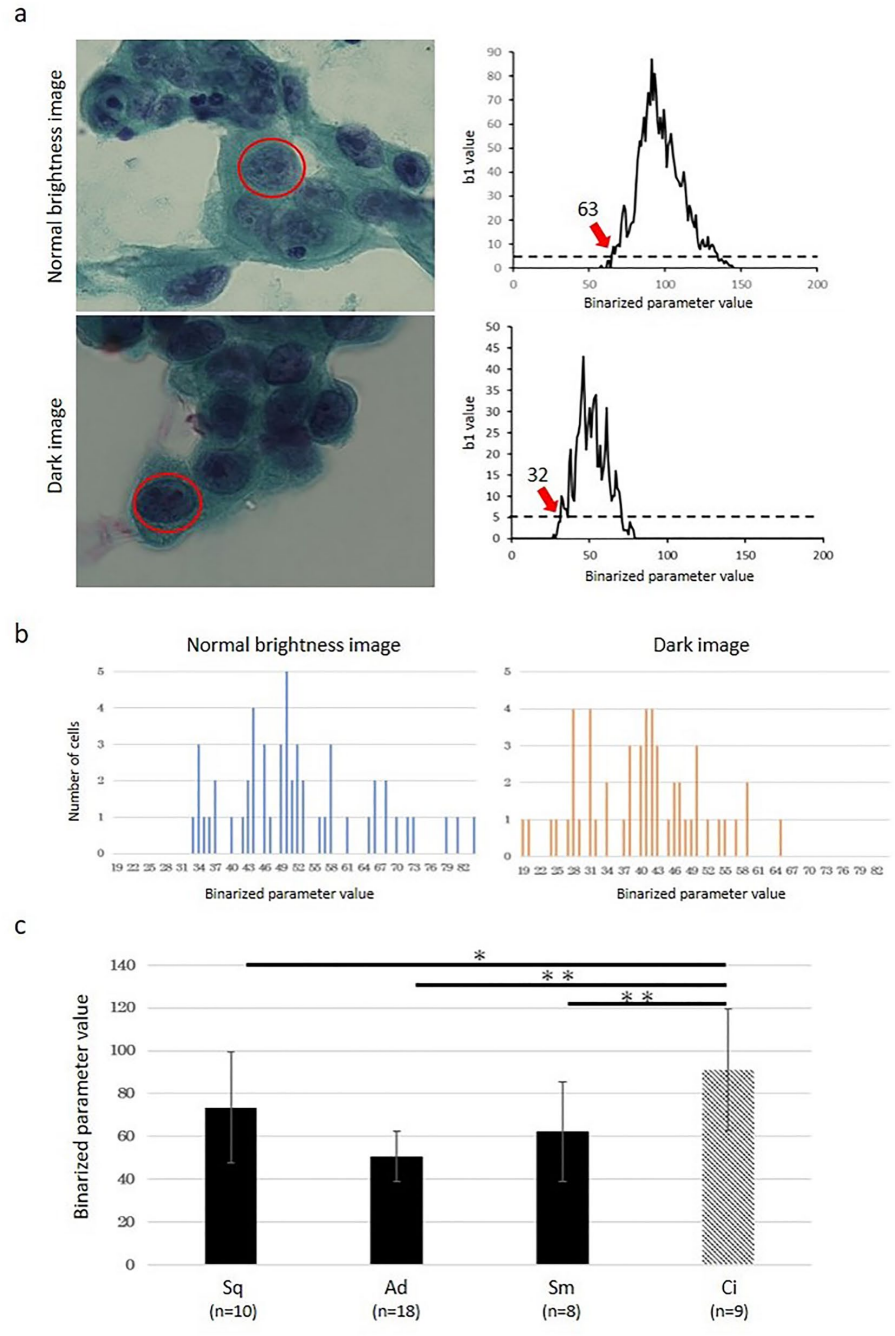
Comparison of the HV in an image with normal brightness versus a dark image. (**a**) The HV was higher for the bright images and lower for darker images. (**b**) The distribution of the HV in 52 adenocarcinoma cells. The distribution of the HV values for the images with normal brightness versus the dark images was 33–84 (median 50) and 19–65 (median 41), respectively. (**c**) A comparison of the HV after median correction across histological types. There was a significant difference between cancer and non-cancerous cells (Student's t test; *P < 0.05, **P < 0.01). Data are shown as the mean ± SD. *Sq* squamous cell carcinoma, *Ad* adenocarcinoma, *Sm* small cell carcinoma, *Ci* ciliated columnar epithelial cell.

The results are shown in Fig. 1. There was a significant difference between non-cancerous and adenocarcinoma (*P* < 0.01), but there were no significant differences found between non-cancerous and squamous carcinoma, or non-cancerous and small cell carcinoma. We also investigated the possibility that the brightness of each image might affect the HV, and found that the HV of darker images tended to have a low HV compared with brighter images (Fig. 2a). The distribution of the HV values for an image with normal brightness (52 adenocarcinomas) was 33–84 (median 50), and that for a dark image (52 adenocarcinomas) was 19–65 (median 41), suggesting that the “holes” in the dark image were more likely to form at a lower stage of binarization (Fig. 2b). Therefore, we normalized the dark images by adding 9, which was the difference of the median values. As a result, the HV of squamous cell carcinoma, adenocarcinoma, and small cell carcinoma was significantly lower than non-cancerous cells (Fig. 2c). Next, we compared the median value of the binarized parameter, b1MAX with normal brightness versus darker images (Supplementary Fig. S3a). As a result, the difference in the median value was 15. We normalized these values by adding 15 and found that the HV of squamous cell carcinoma, adenocarcinoma and small cell carcinoma were also significantly lower than non-cancerous cells using this method (Supplementary Fig. S3b).

**Figure 3:**
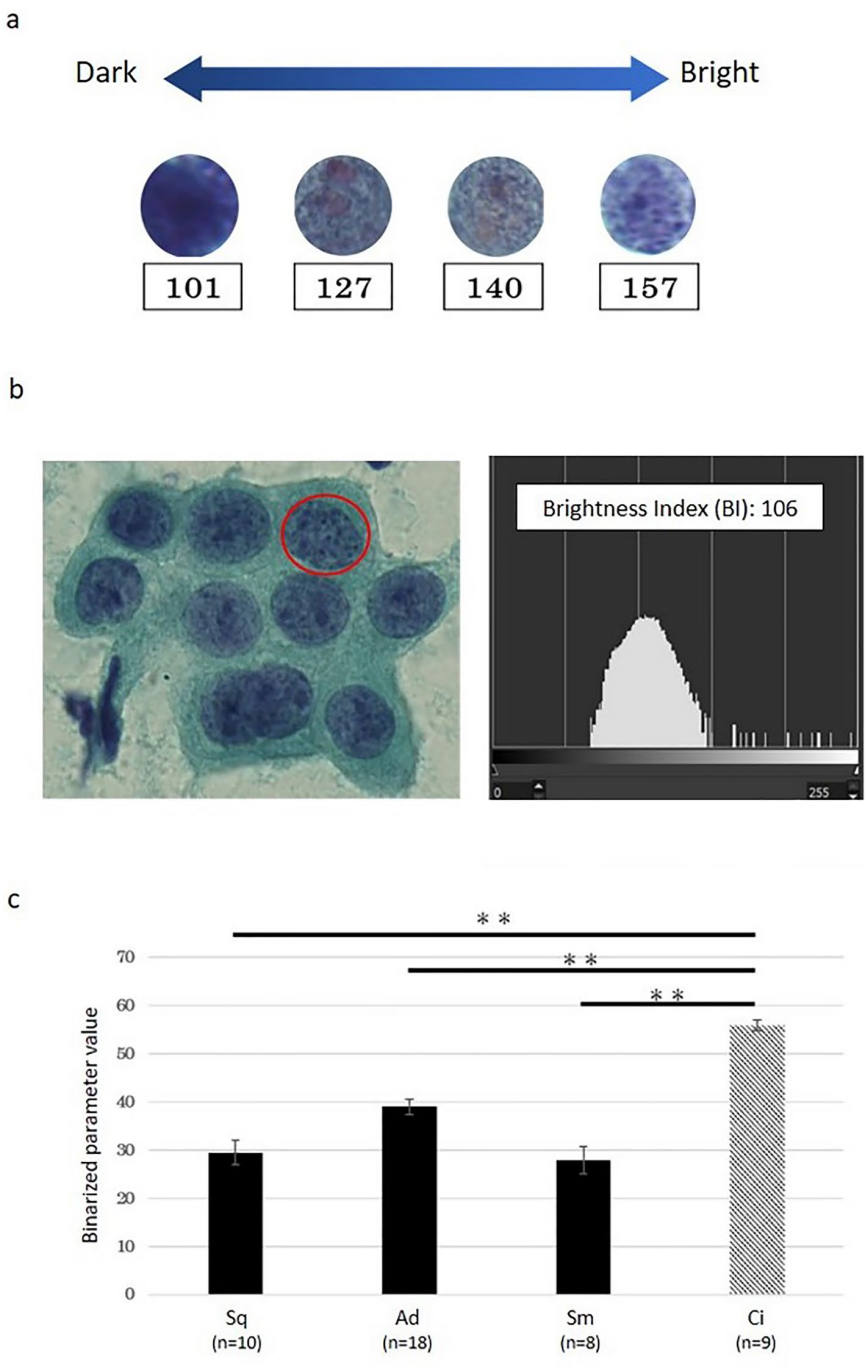
A comparison of the HV after the BI correction. (**a**) The brightness of each nucleus. (**b**) The brightness distribution of adenocarcinoma cells. In this nucleus (red circle), the number of pixels with a brightness of 106 was the highest, and this was defined as the brightness index (BI). (**c**) A comparison of the HV after BI correction across histological types. There was a significant difference between cancer and non-cancerous cells (Student's t test; **P < 0.01). Data are shown as the mean ± SD. *Sq* squamous cell carcinoma, *Ad* adenocarcinoma, *Sm* small cell carcinoma, *Ci* ciliated columnar epithelial cell.

For a different normalization method, we normalized all of the images based on the brightness. Brightness is the value between 0 and 255 with a lower number indicating dark and a higher number indicating bright (Fig. 3a). We calculated the distribution of brightness for each nucleus and defined this as the brightness index (BI), which is the value having the highest pixel number (Fig. 3b). We adjusted the BI of each cell to 127, which was the median value of the brightness and re-analyzed the average HV of squamous carcinoma (10 cases), adenocarcinoma (18 cases), small cell carcinoma (8 cases) and non-cancerous (9 cases). As a result, the HVs of cancer cells, not only adenocarcinoma but also squamous carcinoma and small cell carcinoma were significantly lower than that of non-cancerous cells (*P* < 0.01) (Fig. 3c).

### Comparison of the b1MAX in lung cancer cells versus non-cancerous cells

**Figure 4:**
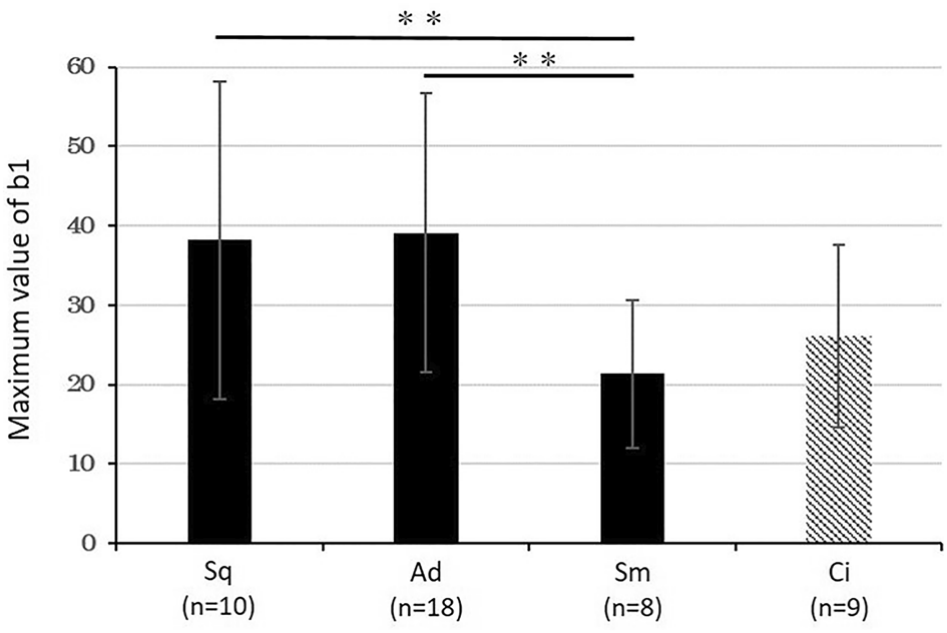
A comparison of the b1MAX value across histological types. There was a significant difference between non-small cell carcinoma and small cell carcinoma (Student's t test; **P < 0.01). Data are shown as the mean ± SD. *Sq* squamous cell carcinoma, *Ad* adenocarcinoma, *Sm* small cell carcinoma, *Ci* ciliated columnar epithelial cell.

The results are shown in Fig. 4. The b1MAX of small cell carcinoma was significantly lower than that of squamous cell carcinoma and adenocarcinoma (*P* < 0.01). This suggests that the b1MAX may be useful to differentiate non-small cell carcinoma from small cell carcinoma. However, no significant differences were found between adenocarcinoma and squamous cell carcinoma.

### Differentiation of histological types by "cell checker" analysis

**Figure 5:**
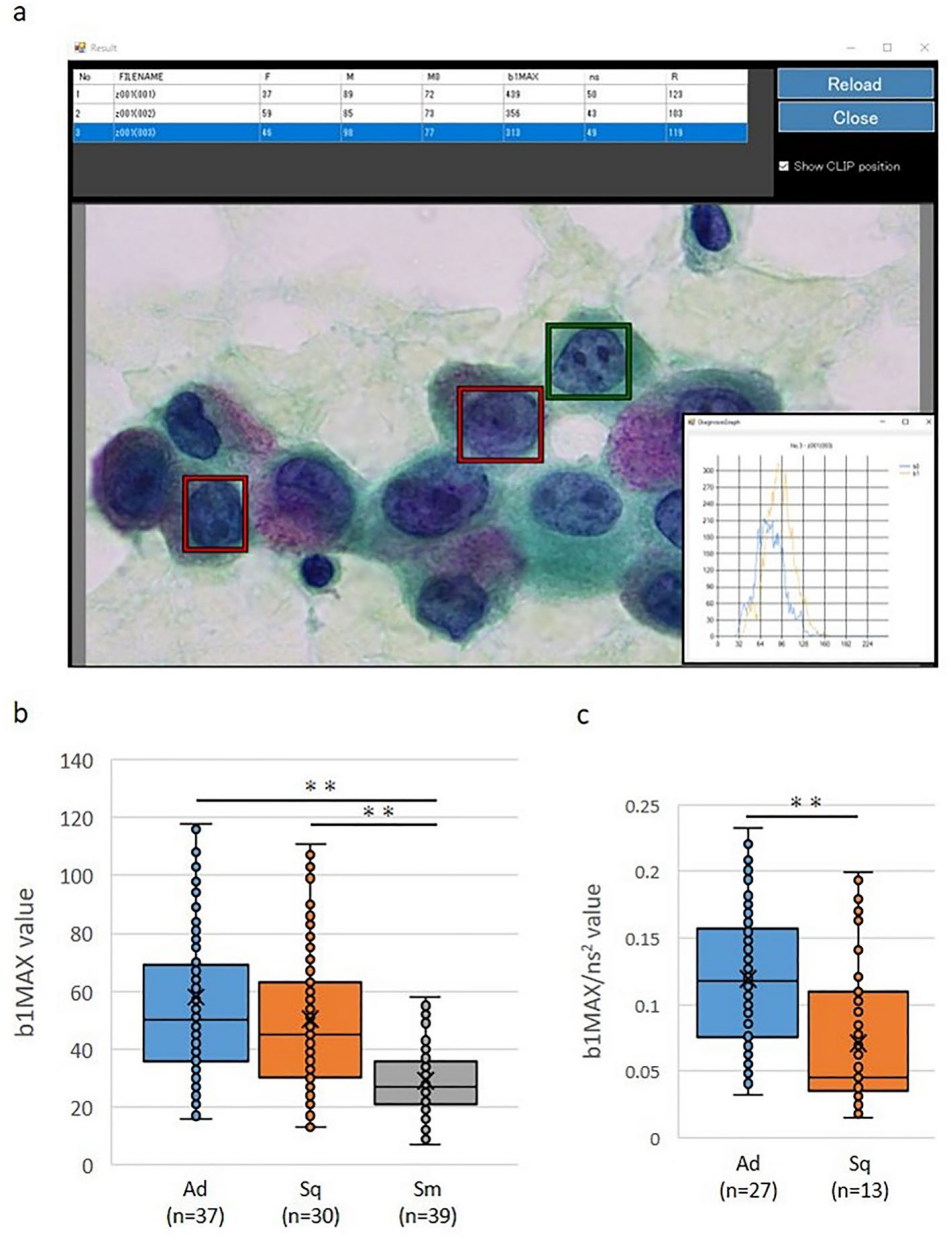
The differentiation of lung cancer histological types by Cell Checker. (**a**) An analysis from a screen by Cell Checker. After the analysis, the b1MAX and ns values were displayed. (**b**) A comparison of the b1MAX across histological types using Cell Checker. There was a significant difference between non-small cell carcinoma and small cell carcinoma (Mann–Whitney’s *U* test; **P < 0.01). *Sq* squamous cell carcinoma, *Ad* adenocarcinoma, *Sm* small cell carcinoma. (**c**) A comparison of the chromatin density in squamous cell carcinoma and adenocarcinoma. There was a significant difference between squamous cell carcinoma and adenocarcinoma (Mann–Whitney’s U test; **P < 0.01). *Sq* squamous cell carcinoma, *Ad* adenocarcinoma.

We investigated the utility of Cell Checker to differentiate the histological types of lung cancer using specimens imaged on microscopes from other institutions (Fig. 5a). The mean b1MAXs for adenocarcinoma, squamous cell carcinoma and small cell carcinoma were 57.96, 50.44 and 28.96, respectively (Fig. 5b). There was a significant difference between non-small cell carcinoma and small cell carcinoma (*P* < 0.01), with a sensitivity and specificity of 72.0% and 71.2%, respectively, and a cutoff of 25.

Next, we attempted to differentiate adenocarcinoma from squamous cell carcinoma by the chromatin density using a high-resolution camera. Since this tool cannot measure area, the lengths of the diagonals of the extracted areas (= ns) were measured with Cell Checker, and the approximate area was calculated by squaring the diagonal lengths (ns^2^). The chromatin density was calculated (b1MAX/ns^2^) by dividing the b1MAX by the ns^2^ value. The result indicates that the b1MAX/ns^2^ of adenocarcinoma was significantly higher than that of squamous cell carcinoma (*P* < 0.01) (Fig. 5c), with a sensitivity and specificity of 80.6% and 90.7%, respectively, and a cutoff of 0.05.

## Discussion

Since the size of the nucleus is enlarged in cancer cells, some studies for automated cytology have focused on nuclear size or the nuclear/cytoplasmic ratio^10, 11, 19^. However, increased chromatin and a heterogeneous chromatin pattern are also morphological hallmarks of cancer cells. In this study, we aimed to develop a homology-based method for the evaluation of the chromatin pattern in cancer and non-cancerous cells. We considered the chromatin as a dot and used Betti numbers, which is part of the theory for homology. In cancer cells, the chromatin is increased and fills in the nucleus. Therefore, we considered that the “hole” (b1) was easily generated by increasing the binarized parameter. In fact, we showed that the HV, which was defined as the parameter of a binarized image where the value of b1 exceeded 5 for the first time, was significantly lower in cancer cells than non-cancerous cells (Figs. 2c, 3c). This finding indicates that the HV may be useful for the determination of cells as cancer versus non-cancerous cells. However, we found that the HV was affected by the image brightness (Fig. 2a,b). This is a clinical challenge for the future because staining methods, reagents and imaging devices are variable among institutions. Therefore, the application of a normalization method for the images is necessary. In this study, we normalized by the median value of the brightness and achieved the differentiation of cancer versus non-cancerous cells (Fig. 3c). Although the specimens used in this study were obtained from one institute, a comparison among multiple institutions will be required, and the other parameters including the level of brightness of the cytoplasm and background may need to be investigated.

**Figure 6:**
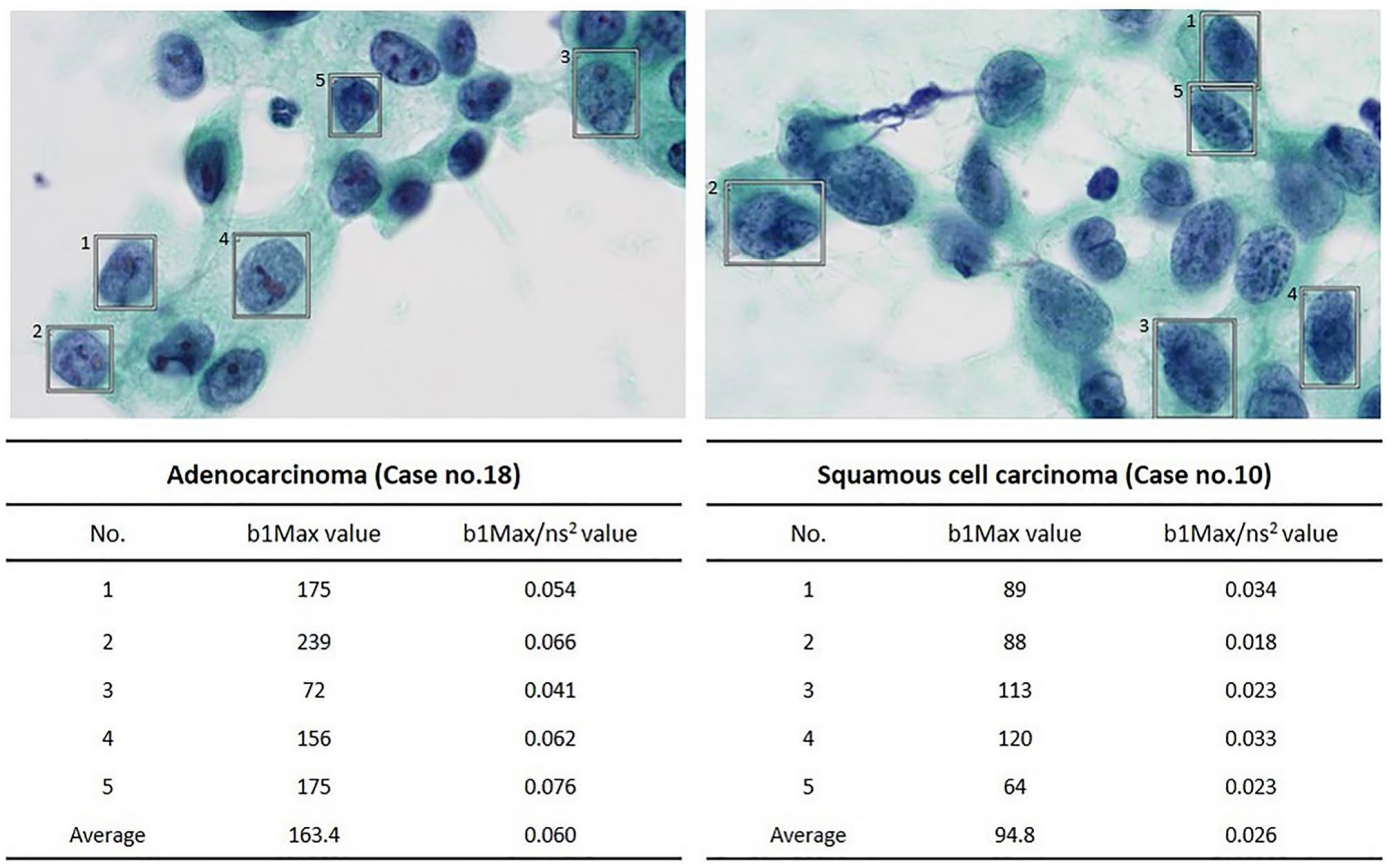
The differentiation between adenocarcinoma and squamous cell carcinoma. Differences in the b1MAX/ns^2^ values were observed, although the morphological differentiation was difficult.

It is important to distinguish between non-small cell carcinoma and small cell carcinoma because the treatment strategy and prognosis is different^5–8^. In the present study, we found a significantly lower b1MAX in small cell carcinoma than in non-small cell carcinoma (Fig. 4). This finding suggests that the b1MAX may be useful to differentiate non-small cell carcinoma from small cell carcinoma. We suppose that the chromatin in small cell carcinoma is highly increased in the form of granules compared to non-small cell carcinoma, therefore, the hole (b1) “disappeared” and the b1MAX was decreased. In addition, unlike the HV, the b1MAX was not affected by the image brightness, so we developed a Cell Checker that automatically analyzes the b1 and ns values (cf. Figs. 2 and 3), and we showed that Cell Checker was useful as a supporting system to differentiate between small cell carcinoma and non-small cell carcinoma or adenocarcinoma and squamous cell carcinoma (Fig. 5b,c). In particular, the chromatin density values calculated by the b1MAX/ns^2^ of adenocarcinomas were significantly higher than those of squamous cell carcinomas, reflecting the fine granular chromatin (Fig. 5c). Since the differences in the b1MAX/ns^2^ values were observed even in cases that were difficult to differentiate microscopically (Fig. 6), the assessment of chromatin density by Cell Checker may play an important role in the selection of molecular targeted therapy. In the future, it will be necessary to develop a tool that can automatically measure area to further improve the accuracy.

**Figure 7:**
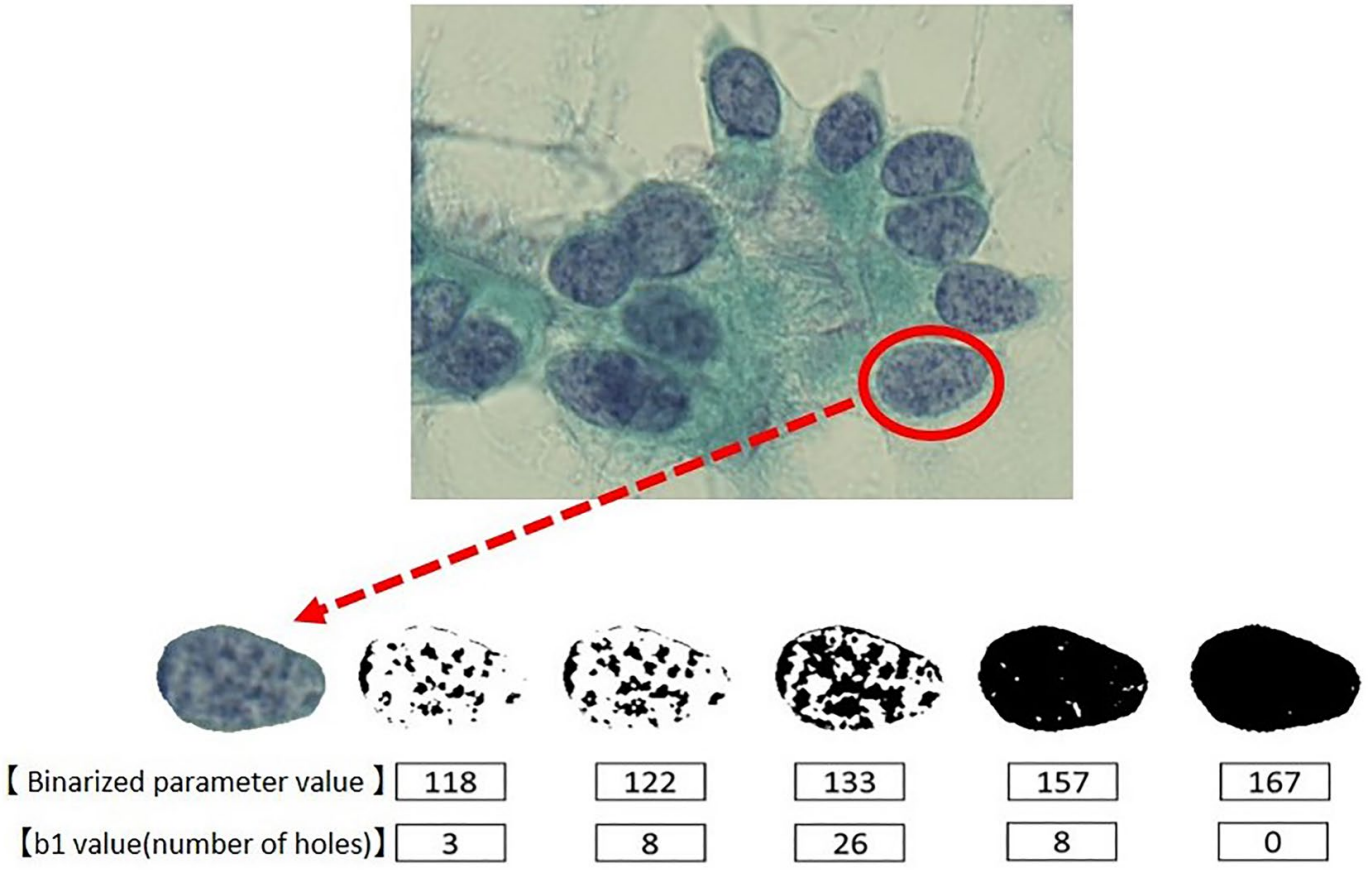
The binarized parameter and b1 value in the CHP method. The nuclei of ciliated columnar epithelial cells were extracted and the binarized parameters were varied. The chromatin was represented by black regions, and the b1 value changed with variability of the binarized parameters.

**Figure 8:**
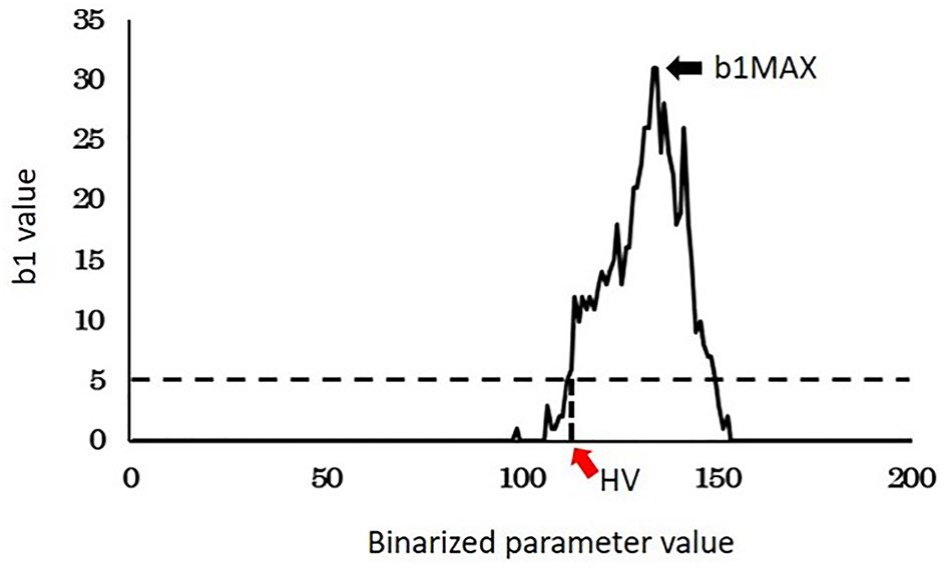
The b1MAX and HV of the CHP method. The b1 values for each parameter were plotted. The maximum value of the b1 was defined as the b1MAX (black arrow), and the binarized parameter with b1 ≥ 5 was defined as the homology value, (red arrow).

According to the principle of autofocus, the brightness of the focused object is highest (Supplementary Fig. S4). When changing the binarization parameters, the chromatin substances on the focal plane first appear as black areas. Subsequently, chromatin substances deviating from the focal plane above and below gradually appear as overlapped areas. Specifically, these are the binarized images in Fig. 7. Ultimately, it is possible to obtain three-dimensional information about chromatin distributed above and below the focal plane. Although exact differential geometric information has been lost, due to the topological invariant, the topological information is partially preserved. The homology profiles in Fig. 8 do not have the complete 3D representation, however, they contain a certain level of 3D information. Confirming whether the obtained 3D information is sufficient for diagnosis constitutes the main result of this paper.

In this study, it might be a very thin thickness, we have carried out an analysis of images projecting the 3D distribution of chromatin within the cell nucleus onto 2D planes. To precisely assess the chromatin distribution, it would be necessary to use the index b2 or hyperedges. However, considering the amount of data and computational power required for that, it is not practical.

Also, the homology features (such as b1MAX) could be correlated with tumor genome characteristics. We have obtained some related results, but further examination is necessary, making it a topic for future research. Furthermore, as the automation of the currently developed tool progresses or if it is implemented online, it can be utilized in various facilities. This is also a matter we aim to explore in our future research as well.

In summary, we showed that the chromatin homology profile method is an available tool for respiratory cytology. Notably, the b1MAX and b1MAX/ns^2^ values are potentially useful for differentiating histological types of lung cancer. By combining the CHP method and other approaches, we expect to further improve the automatic detection of lung cancer and the differentiation of histological types.

## The concept of the algorithm

### Chromatin homology profile (CHP) method

Homology is a mathematical tool to quantify "the contact degree", which is expressed in terms of Betti numbers. The Betti numbers for the CHP method described here, consisted of two numbers, b0 (a zero-dimensional Betti number) and b1 (a one-dimensional Betti number) (Supplementary Fig. S1). The chromatin homology profile method quantifies the chromatin contact degree and detects differences in chromatin patterns. In this study, the b1 value, or the number of “holes” (surrounded areas), formed by chromatin contact, was mainly used.

## Materials and methods

Forty-five respiratory cytology specimens (squamous cell carcinoma 10 cases, adenocarcinoma 18 cases, small cell carcinoma 8 cases and lung non-cancerous cells 9 cases), diagnosed at Osaka University Hospital were included. For the image cytology, we used a high-performance microscope BZ-X700 (KEYENCE), which acquires multilayer (z-stacking) images (Supplementary Fig. S2). We selected 3 to 4 fields from each slide and acquired 20 to 30 pictures with 0.5 µm interval per field using the 100 × objective. Among them, we selected 3 to 4 cells with nuclei that were clearly visible from each field. In total, we analyzed 7 to 16 nuclei per slide. The nucleus extraction was performed manually using drawing software (GIMP). The extracted nucleus was binarized in 256 steps from 0 to 255, and the b1 value for each binarized parameter value was counted (Fig. 7). After counting, the change in the b1 value was plotted, and the maximum value (b1MAX) was calculated. In addition, we defined the homology value (HV) as the parameter of the binarized image where the value of b1 exceeded 5 for the first time (Fig. 8). We compared the HV and b1MAX in squamous cell carcinoma, adenocarcinoma, small cell carcinoma and non-cancerous cells.

Furthermore, we developed an analysis application "Cell Checker" and examined its utility to differentiate histological types of lung cancer using specimens acquired on microscopes from other institutions. A total of 146 cases (adenocarcinoma 64 cases, squamous cell carcinoma 43 cases and small cell carcinoma 39 cases) with lung cancer diagnosed at Habikino Medical Center between 2017 and 2021 were used in this study. Papanicolaou stained specimens were acquired by microscope BX43 (OLYMPAS) and ECLIPSE Ci-E (Nikon), and 3 to 7 cancer cells per case were acquired with a 100 × objective by low- (Visualix Pro2Metrics, Visualix) and high-resolution cameras (4 K Tribrid Camera, BioTools). This study was approved by the Ethics committee of Osaka University Hospital (approval no. 19390) and Habikino Medical Center (approval no. 1109), and complied with the Declaration of Helsinki, and informed consent were obtained from all participants.

### Statistical analysis

The data were compared by Student's t-test and Mann–Whitney’s *U* test. A value of *P* < 0.05 was considered statistically significant. All statistical analyses were performed using Microsoft Excel or Statcel 4.

### Supplementary Information

Supplementary Legends.

Supplementary Figure S1.

Supplementary Figure S2.

Supplementary Figure S3.

Supplementary Figure S4.

## Data Availability

The data generated in this study are available upon request without restriction from the corresponding author.
