# Supplementary material for: A quantitative evaluation method utilizing the homology concept to assess the state of chromatin within the nucleus of lung cancer
Source: Sci Rep. 2023 Nov 9;13:19585. doi: 10.1038/s41598-023-46213-w (PMC10638289; doi:10.1038/s41598-023-46213-w)
Supplement: Supplementary file 1 — Supplementary Legends. [file 41598_2023_46213_MOESM1_ESM.docx]

**Supplementary information**

Supplementary Fig. S1. Betti number. The number of independent figures is denoted by b0, and the number of areas enclosed by a figure is represented by b1. In the left figure, b0=7, b1=0; in the right figure, b0=2, b1=3.

Supplementary Fig. S2. Images of squamous cell carcinoma and adenocarcinoma acquired with a 100x objective. a squamous cell carcinoma (Pap. ×100). b adenocarcinoma (Pap. ×100). The nuclear chromatin was confirmed in detail using a 100x objective (oil immersion).

Supplementary Fig. S3. A comparison of the binarized parameter value for the b1MAX in images with normal brightness versus dark images. a The distribution of the HV for 52 adenocarcinoma cells. The distribution of the HV values for images with normal brightness and dark images was 38-104 (median 74.5) and 34-93 (median 59.5), respectively. b A comparison of the HV after the median correction for histological types. There was a significant difference between cancer and non-cancerous cells (Student's t test; **P* < 0.05, ***P* < 0.01). Data are shown as the mean ± SD. Sq; squamous cell carcinoma, Ad; adenocarcinoma, Sm; small cell carcinoma, Ci; ciliated columnar epithelial cell.

Supplementary Fig. S4. A schematic illustration viewed from the side of a cell. The solid line represents the focal plane. According to the principle of autofocus, the brightness of the focused object is highest. Therefore, when changing the binarization parameters, the chromatin substances on the focal plane first appear as black areas. Subsequently, chromatin substances deviating from the focal plane above and below gradually appear as overlapped areas. Specifically, these are the binarized images in Fig. 7.
