# Supplementary figures and images for: A quantitative evaluation method utilizing the homology concept to assess the state of chromatin within the nucleus of lung cancer
Source: Sci Rep. 2023 Nov 9;13:19585. doi: 10.1038/s41598-023-46213-w (PMC10638289; doi:10.1038/s41598-023-46213-w)

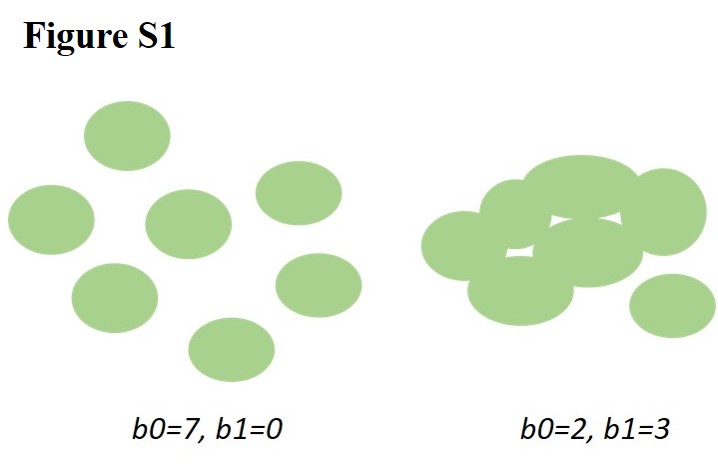

Supplement: Supplementary file 2 — Supplementary Figure S1. [file 41598_2023_46213_MOESM2_ESM.jpg]

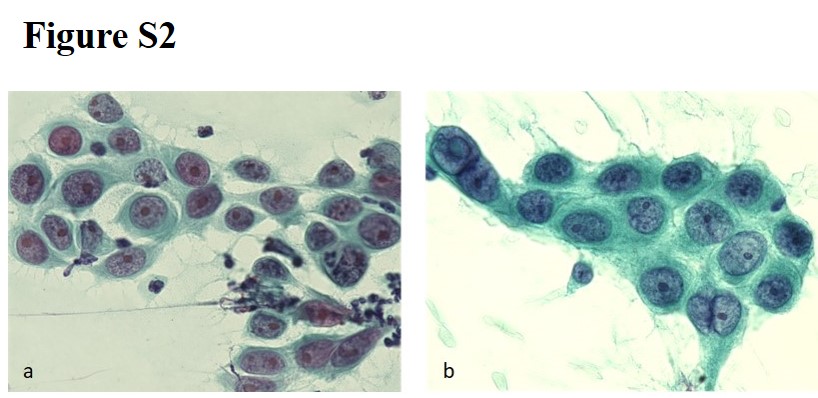

Supplement: Supplementary file 3 — Supplementary Figure S2. [file 41598_2023_46213_MOESM3_ESM.jpg]

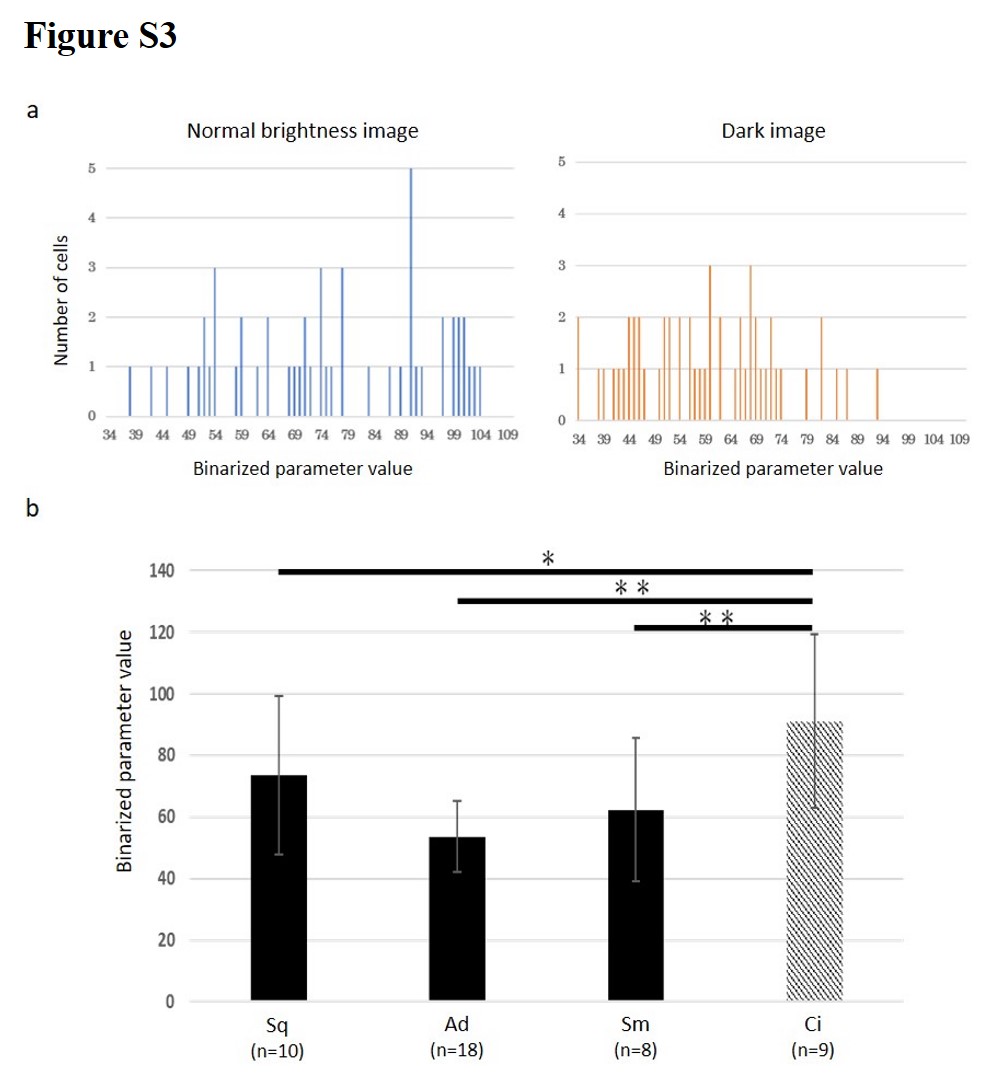

Supplement: Supplementary file 4 — Supplementary Figure S3. [file 41598_2023_46213_MOESM4_ESM.jpg]

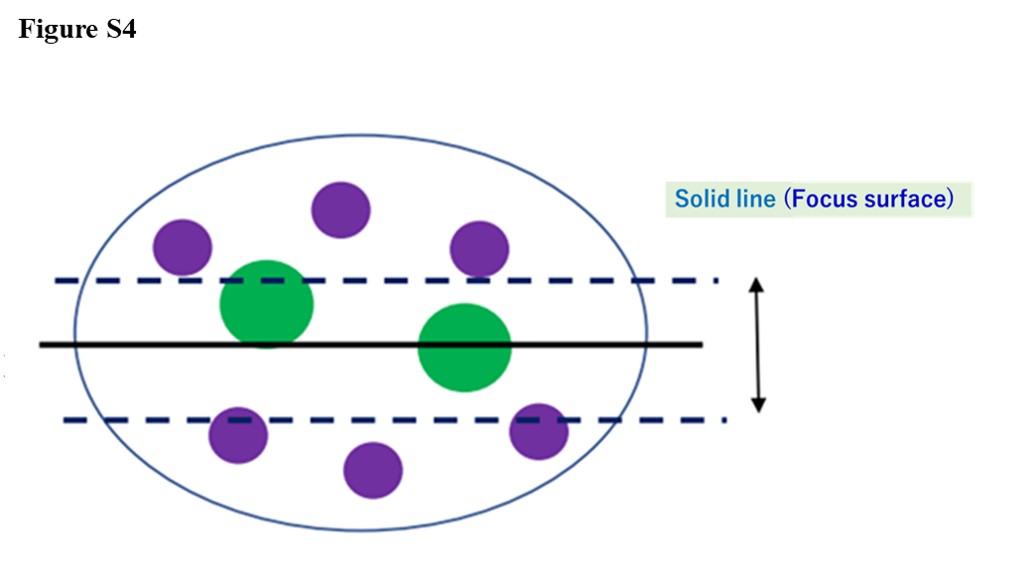

Supplement: Supplementary file 5 — Supplementary Figure S4. [file 41598_2023_46213_MOESM5_ESM.jpg]
